# In-hospital and long-term clinical outcomes of spontaneous coronary artery dissection (SCAD): a meta-analysis of conservative versus revascularization approaches

**DOI:** 10.1186/s43044-024-00585-0

**Published:** 2024-11-22

**Authors:** Anmol Pitliya, Aakanksha Pitliya, Srivatsa Surya Vasudevan, Kumari Priya Yadav, Muhammad Bilal Shabbir, Shaghaf Zahoor, Aisha Shabbir, Abdulgafar Dare Ibrahim, Bijay Mukesh Jeswani, Ramya Reddy Jonnala, Ramit Singla

**Affiliations:** 1 Department of Hospitalist Medicine, Camden Clark Medical Center, Parkersburg, WV USA; 2 Department of Internal Medicine, Mercy Catholic Medical Center, Darby, PA USA; 3 Department of Otorhinolaryngology- Head and Neck Surgery, Louisiana State University Health Center, Shreveport, LA USA; 4 Department of Medicine, Goa Medical College and Hospital, Goa, India; 5 Department of Medicine, Army Medical College, Rawalpindi, Pakistan; 6 Department of Medicine, Army Medical College, Rawalpindi, Pakistan; 7 Department of Medicine, King Edward Medical University, Lahore, Pakistan; 8 Department of Internal Medicine, Piedmont Atlanta Hospital, Atlanta, GA USA; 9 Department of Medicine, GCS Medical College and Hospital, Ahmedabad, India; 10 Department of Medicine, AIIMS, Bhopal, India; 11 Department of Neurology, Medical University of South Carolina, Columbia, SC USA

**Keywords:** Coronary artery dissection, Coronary disease, Ischemic heart disease, Spontaneous coronary artery dissection, Dissected coronary artery

## Abstract

**Background:**

The ideal treatment strategy for spontaneous coronary artery dissection (SCAD) remains unclear, with patients potentially treated with either conservative medical care or a revascularization approach.

**Methods:**

We performed a systematic review and meta-analysis adhering to PRISMA 2020 guidelines. Inclusion criteria involved studies with confirmed SCAD diagnosis, reporting initial management strategies, and original research with ≥ 10 participants. Random-effect models were applied for insignificant heterogeneity with significance at *p* ≤ 0.05. Sensitivity analysis and funnel plots assessed potential publication bias.

**Results:**

Our analysis found no significant differences in major adverse cardiac events (MACE) (OR = 0.61, *p* = 0.49), unstable angina pectoris (UAP) (OR = 1.04, *p* = 0.93), non-ST segment elevation myocardial infarction (NSTEMI) (OR = 1.16, *p* = 0.82), recurrent myocardial infarction (MI) (OR = 0.78, *p* = 0.56), stroke (OR = 0.35, *p* = 0.07), heart failure (OR = 0.41, *p* = 0.24), in-hospital mortality (OR = 0.35, *p* = 0.09), post-discharge mortality (OR = 1.66, *p* = 0.27), or ST segment elevation myocardial infarction (STEMI) (OR = 0.45, *p* = 0.23) between conservative management and revascularization procedures. However, sensitivity analysis reveals significant decreases in odds of inferior wall STEMI (OR = 0.41 [95% CI 0.17–0.97], *p* = 0.04) and heart failure (OR = 0.18 [95% CI 0.06–0.54], *p* = 0.002) in conservative treatment compared to revascularization group.

**Conclusion:**

Conservative therapy significantly decreased inferior wall STEMI and heart failure as compared to revascularization in SCAD. Although no significant differences in cardiovascular outcomes, sensitivity analysis highlights potential benefits of conservative management.

**Supplementary Information:**

The online version contains supplementary material available at 10.1186/s43044-024-00585-0.

## Background

Spontaneous coronary artery dissection (SCAD) is an occasional cause of acute coronary syndrome, predominantly affecting younger women, characterized by the spontaneous separation of an epicardial coronary artery wall and the concomitant formation of an intramural hematoma (IMH) leading to myocardial ischemia [1]. Two theories on SCAD's formation include the inside-out hypothesis, suggesting an intimal tear as the primary event leading to a false lumen, and the outside-in hypothesis, proposing a primary disruption of vasa vasorum causing bleeding into the vessel wall and subsequent intimal rupture in certain patients [2, 3]. Formerly regarded as an uncommon condition, SCAD has gained significance as a notable contributor to acute coronary syndrome, particularly among young women.

SCAD constitutes up to 4% of all cases of acute coronary syndrome (ACS) and is identified in 0.02–1.10% of coronary angiographies [4–20]. While SCAD remains relatively infrequent in the general population as a cause of myocardial infarction (MI), its prevalence significantly rises in ACS cohorts of younger individuals, reaching 3.1–9.7% in patients with premature MI under 45 years of age [21–23]. Among younger females, SCAD accounts for 8.8–11.1% of ACS events in those less than 60 years old and 8.7–45.0% in women under 50 years of age. 24–25 Additionally, SCAD is notably prominent in pregnancy-associated MI, representing 43% of cases, with a prevalence of 2 cases per 100,000 pregnancies in Canada, although accounting for 4.7–16.7% of overall SCAD cases [23–29].

While the clinical manifestations of SCAD may resemble atherosclerotic acute coronary syndrome ACS, SCAD exhibits a more favorable prognosis, particularly in patients without ongoing ischemia or hemodynamic instability [30, 31]. The management of SCAD remains contentious, as there is currently no universally accepted gold standard approach, and the effectiveness of specific medical regimens or coronary revascularization techniques in comparison to conservative medical management is not well-established. Recent research indicates that among patients initially treated conservatively, 73–100% experience spontaneous healing of dissected lesions upon subsequent angiography, whereas the revascularization approach with percutaneous coronary intervention (PCI) or coronary artery bypass grafting (CABG) for SCAD patients is constrained by a significant risk of procedural complications or conduit failure [31, 32]. Presently, conservative therapy is generally favored over revascularization for stable SCAD patients lacking high-risk features; however, the comparative benefits in terms of protection against subsequent ischemia, SCAD recurrence, heart failure, and mortality remain uncertain [2].

This meta-analysis aims to assess the comparative clinical outcomes between conservative management with medical therapy and revascularization procedures with PCI or CABG in patients with SCAD drawing insights from published data.

## Methods

We carried out a systematic review and meta-analysis using the Preferred Reporting Items for Systematic Reviews and Meta-analysis (PRISMA 2020) guidelines [32].

### Eligibility criteria for considering studies under this review

#### Inclusion criteria

The studies were chosen for inclusion based on the following participant, intervention, and outcome characteristics. Population: Patients with a confirmed diagnosis of SCAD based on angiography. Intervention: The initial management with conservative therapy. Comparison: revascularization strategies (PCI or CABG). Outcomes: In-hospital and long-term clinical outcomes.

The inclusion criteria for the identification of relevant studies are as follows: Participants must have a confirmed diagnosis of SCAD based on angiography, the study's findings must be presented as an original research article, excluding case reports, reviews, editorials, or commentaries, the study must provide information on the initial management strategy employed for SCAD, the study must report clinical outcomes, encompassing both in-hospital and/or long-term results, the sample size of the study should be equal to or greater than 10 participants. Moreover, the review specifically encompassed studies published in English focused exclusively on the adult population (aged > 19 years) and involved human participants.

#### Exclusion criteria

The exclusion criteria for the identification of relevant studies are as follows: studies where participants lack a confirmed diagnosis of SCAD based on angiography, studies presenting findings in the form of case reports, reviews, editorials, or commentaries, studies lacking information on the initial management strategy employed for SCAD, studies not reporting management strategies, and studies with a sample size less than 10 participants. Additionally, studies not published in English, not exclusively focused on the adult population (aged > 19 years), or not involving human participants were excluded from the review.

### Search strategy for identification of records in this review

The search strategy employed for this study involved a systematic exploration of the Ovid MEDLINE database (1946 to present), PubMed (1947 to present), and the Embase database (1947 to present). The search utilized relevant keywords and medical subject headings (MeSH) related to SCAD. The specific terms included “SCAD,” “dissected coronary artery,” and “coronary dissection.” We also alphabetized all references using EndNote to eliminate duplicates. A total of 3026 studies were found using three databases. After deleting 1540 duplicate articles, we reviewed the remaining 1486 articles based on their titles and abstracts. Only articles written in English till January 2024 have been identified through the entire database search.

### Selection of studies for inclusion in the review

Initially, citations underwent a title/abstract level screening by two independent reviewers, and complete manuscripts were obtained if deemed potentially relevant. Disagreements were resolved through consensus. The same reviewers independently evaluated identified articles based on the inclusion criteria, specifically focusing on clinical outcomes when comparing conservative and revascularization strategies for SCAD management. Disputes related to inclusion criteria were settled through consensus. Studies comparing the two strategies without reporting clinical outcomes were excluded. Additionally, studies that, even after assessing clinical outcomes, did not provide detailed information on the type of treatment strategy used were also excluded.

### Assessment of the methodological quality and risk of *bias*

The evaluation of the methodological quality of the studies included in the meta-analysis was performed by two researchers. We used the Newcastle–Ottawa Scale (NOS) for case–control and cohort studies based on selection, comparability, and outcomes [33]. Additionally, the Joanna Briggs Inventory (JBI) tool was used for risk of bias assessment for case series [34]. Refer to the supplementary file for details.

### Data extraction and management

The data were extracted into a Microsoft Excel spreadsheet, including details such as the first author, year of publication, country of study, type of study, prevalence of SCAD, sample size, mean age, male and female populations, and specific outcome measures including ACS, ST segment elevation myocardial infarction (STEMI), non-ST segment elevation myocardial infarction (NSTEMI), follow-up duration, and various other parameters to compare outcomes between the conservative and revascularization approaches.

### Statistical analysis

Meta-analyses were conducted using Comprehensive Meta-Analysis (CMA) software version 4, which quantitatively evaluated treatment variables by computing the odds ratio (OR) along with the corresponding 95% confidence interval (CI). Heterogeneity among study results was assessed using Chi-square and I2 statistics. Insignificant heterogeneity (I2 < 50%, *p* > 0.1) was observed, indicating a random-effects model was appropriate. A significance level of *p* ≤ 0.05 was used to determine statistical significance. A sensitivity analysis was performed to assess the stability of the results. Additionally, contour-enhanced funnel plots and Egger’s regression were employed to identify potential publication bias using R statistical software version 4.3.2.

## Results

### Study identification and baseline characteristics

**Fig. 1:**
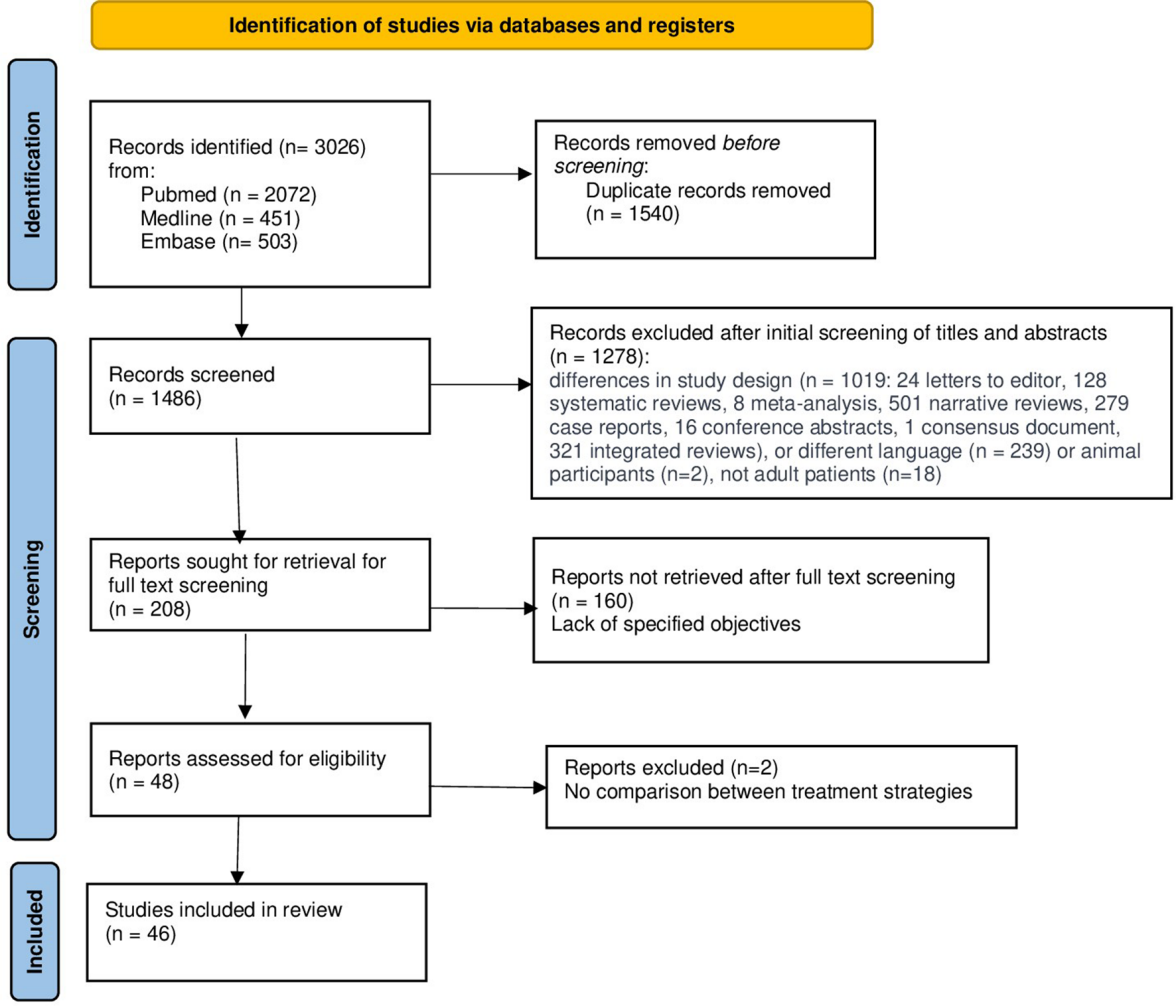
The PRISMA flowchart detailing the identification and screening process used to select the final articles for inclusion in the review

A total of 3026 records were initially identified, of these, 1486 records were considered excluded on screening titles/abstracts. Of these, 1278 studies were further excluded because of study designs other than observational studies, different languages, animal participants, and patients less than 19 years of age. Furthermore, 208 studies were retrieved for full-text screening. We further excluded 160 studies due to a lack of specified objectives. The studies underwent quality assessment using specified tools [33, 34] excluding 2 studies that did not compare the treatment strategies. The review finally included 46 studies, including 30 cohort studies (69.56%), 2 case–control (4.35%), and 14 case series (26.09%) [9–11, 17, 18, 20, 24, 28, 30, 31, 35–70]. The data collection process for the review ended on February 20, 2024. Figure 1 in the review article shows the PRISMA flowchart detailing the identification and screening process used to select the final articles for inclusion in the review.

The study comprised 13,539 SCAD patients. Among them, 5338 (39.4%) underwent conservative treatment, while 8201 (60.5%) underwent a revascularization approach. The study and clinical characteristics are summarized in Table 1. The mean age of participants is 52.63 ± 6.69 years, with an 88% predominance of females. The prevalence of ACS among the study participants was high, recorded at 92.64% Among these ACS cases, nearly half were identified as STEMI, accounting for 44.73% of the total cases, while the remaining cases were classified as NSTEMI with a prevalence of 51.34%. The median (range) follow-up was 30 (3.8–90) months.

**Table 1:** Summary of study characteristics

Included studies	Country of study	Type of study	Mean age, years	Sample size of SCAD patients	Conservative approach	Revascularization approach	Follow-up duration
Mortensen et al. [17]	Denmark	Retrospective cohort	48.7	22	7	15	34.8
Vanzetto et al. [18]	France	Case series	46	23	10	13	15.6
Motreff et al. [35]	France	Case series	43.8	12	2	10	51
Ito et al. [46]	USA	Retrospective cohort	45	23	18	5	39
Kansara et al. [36]	USA	Case series	40.7	13	8	5	12
Alfonso-a et al. [37]	Spain	Prospective cohort	48	11	7	4	17.4
Alfonso-b et al. [30]	Spain	Prospective cohort	53	45	36	9	24.3
Tweet et al. [40]	USA	Retrospective cohort	42.6	87	44	43	47
Saw et al. [31]	Canada	Retrospective cohort	52.1	168	134	29	82.8
Tweet et al. [47]	USA	Retrospective cohort	44	189	94	95	26
Manhaes et al. [38]	Brazil	Case series	48.8	25	14	11	75.6
Lettieri et al. [49]	Italy	Retrospective cohort	52	134	78	56	31
Sultan et al. [28]	USA	Case series	42.3	10	6	4	ND
McGrath-Cadell et al. [39]	Australia	Retrospective cohort	45	40	27	13	16
Nakashima et al. [69]	Japan	Retrospective cohort	46	63	28	35	34
Roura et al. [40]	Spain	Case series	47	34	26	8	4
Godinho et al. [41]	Portugese	Retrospective cohort	51	17	13	4	30
Rashid et al. [42]	Australia	Case series	53.3	23	17	6	ND
Cade et al. [43]	Brazil and USA	Case series	33.8	13	7	6	20.3
Lobo et al. [50]	USA	Retrospective cohort	49.3	51	17	34	12
Rogowski et al. [51]	Switzerland	Prospective cohort	53	64	56	8	54
Saw et al. [52]	Canada	Prospective cohort	52.5	327	272	55	37
Adams et al. [53]	Australia	Case–control	48.7	22	17	5	12
Sharma et al. [54]	USA	Retrospective cohort	46	102	63	39	ND
Mahmoud et al. [55]	USA	Retrospective cohort	61.7	7,347	904	6443	ND
Abreu et al. [44]	Portugal	Case series	56	27	15	12	20
Saw et al. [56]	Canada	Prospective cohort	51.8	750	632	110	36
Clare et al. [10]	USA	Retrospective cohort	49	208	173	32	58
Liu et al. [20]	China	Retrospective cohort	57	118	33	85	43
Inohara et al. [45]	Japan	Case series	52.8	322	147	175	
Seidl et al. [64]	Switzerland	Prospective cohort	53	105	97	8	90
Daoulah et al. [12]	4 Arab Gulf countries (KSA, UAE, Kuwait, and Bahrain)	Retrospective cohort	44	83	33	44	18.8
Díez-Villanueva P et al. [57]	Spain	Prospective cohort	54	318	247	71	ND
McAlister et al. [58]	New Zealand	Case series	ND	117	99	14	ND
Marcos García-Guimaraes et al. [59]	Spain	Prospective cohort	53	318	248	70	ND
Frederic De Roeck et al. [60]	Belgium	Retrospective cohort	40	27	18	9	ND
Chen et al. [29]	USA	Case–control	37.1	307	226	81	68.4
Inoue et al. [24]	Japan	Retrospective cohort	48.7	19	7	12	30
Saw et al. [62]	Canada	Prospective cohort	51.7	750	632	111	36
Salamanca et al. [63]	Spain	Retrospective cohort	54	389	305	84	29
Wilander et al. [65]	Sweden	Retrospective cohort	52.9	147	88	59	17.3
Thaler et al. [66]	USA	Retrospective cohort	49	139	79	60	3.8
Chan et al. [67]	European SCAD registry	Case series	32	82	46	36	ND
Benenati et al. [68]	Spain and Italy	Retrospective cohort	53	375	240	129	ND
Proença et al. [69]	Portugal	Retrospective cohort	51	36	32	4	40
Yuanji Ma et al. [70]	China	Prospective cohort	54.87	81	36	40	12
ND, no data							

### *Meta*-analysis of clinical outcomes of SCAD

**Fig. 2:**
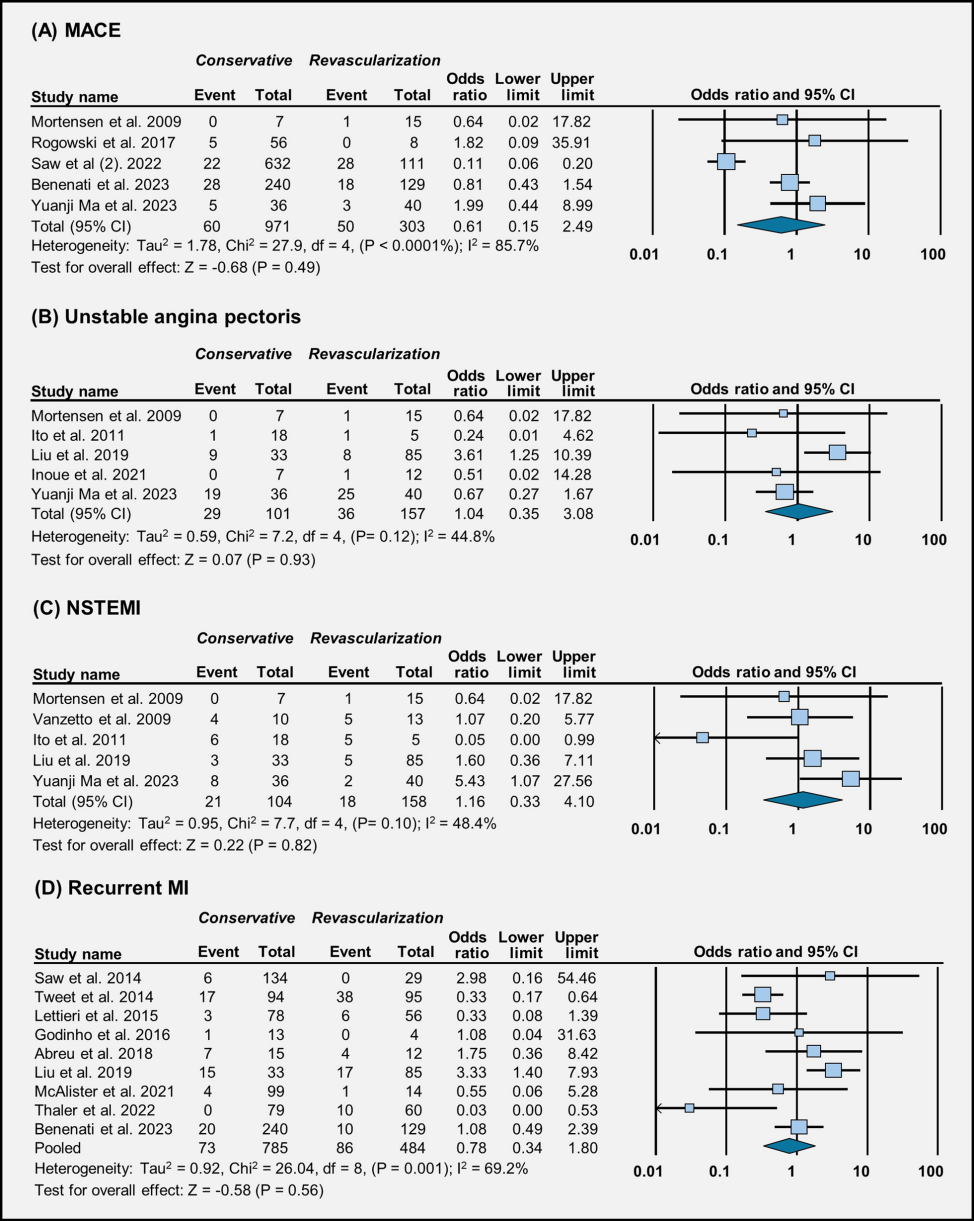
Forest plot illustration of treatment outcomes: **A** MACE, **B** unstable angina pectoris, **C** NSTEMI, and **D** recurrent MI

Our analysis, drawing from data across five studies, suggests that there is no significant difference in major adverse cardiac events (MACE) between conservative management and revascularization procedures (OR = 0.61 [95% CI 0.15–2.49], *p* = 0.49) (Fig. 2A). Across five studies, our examination suggests that the occurrence of unstable angina pectoris (UAP) is similar regardless of whether patients received conservative or revascularization treatment (OR = 1.04 [95% CI 0.35–3.07], *p* = 0.93) (Fig. 2B).

Based on data from five studies, the rate of NSTEMI does not seem to differ much between conservative management and revascularization (OR = 1.16 [95% CI 0.33–4.10], *p* = 0.82) (Fig. 2C). Our analysis spanning nine studies suggest that the risk of recurrent MI appears similar between conservative management and revascularization (OR = 0.78 [95% CI 0.33–1.80], *p* = 0.56) (Fig. 2D).

**Fig. 3:**
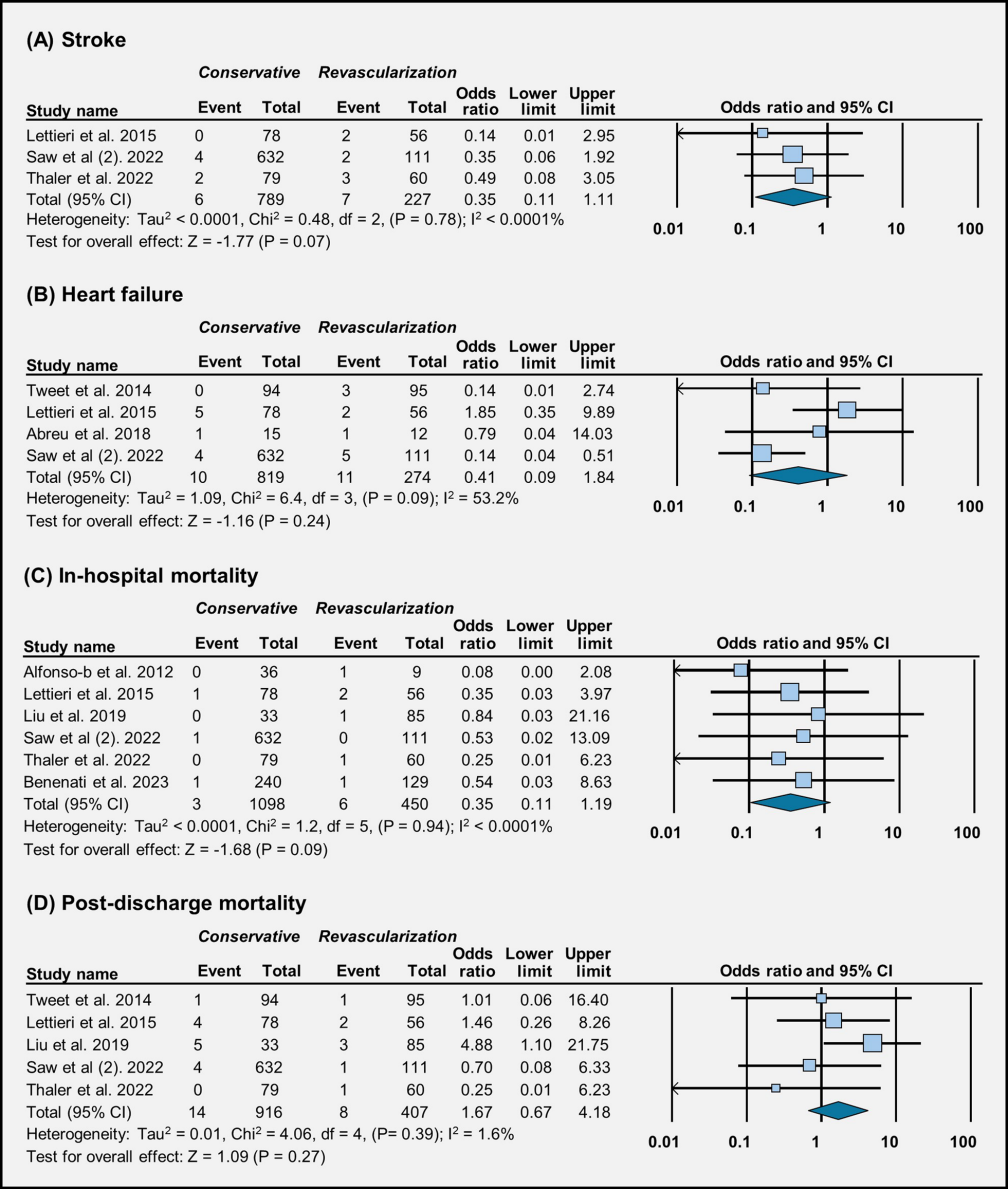
Forest plot illustration of treatment outcomes: **A** stroke, **B** heart failure, **C** in-hospital mortality, and **D** post-discharge mortality

**Fig. 4:**
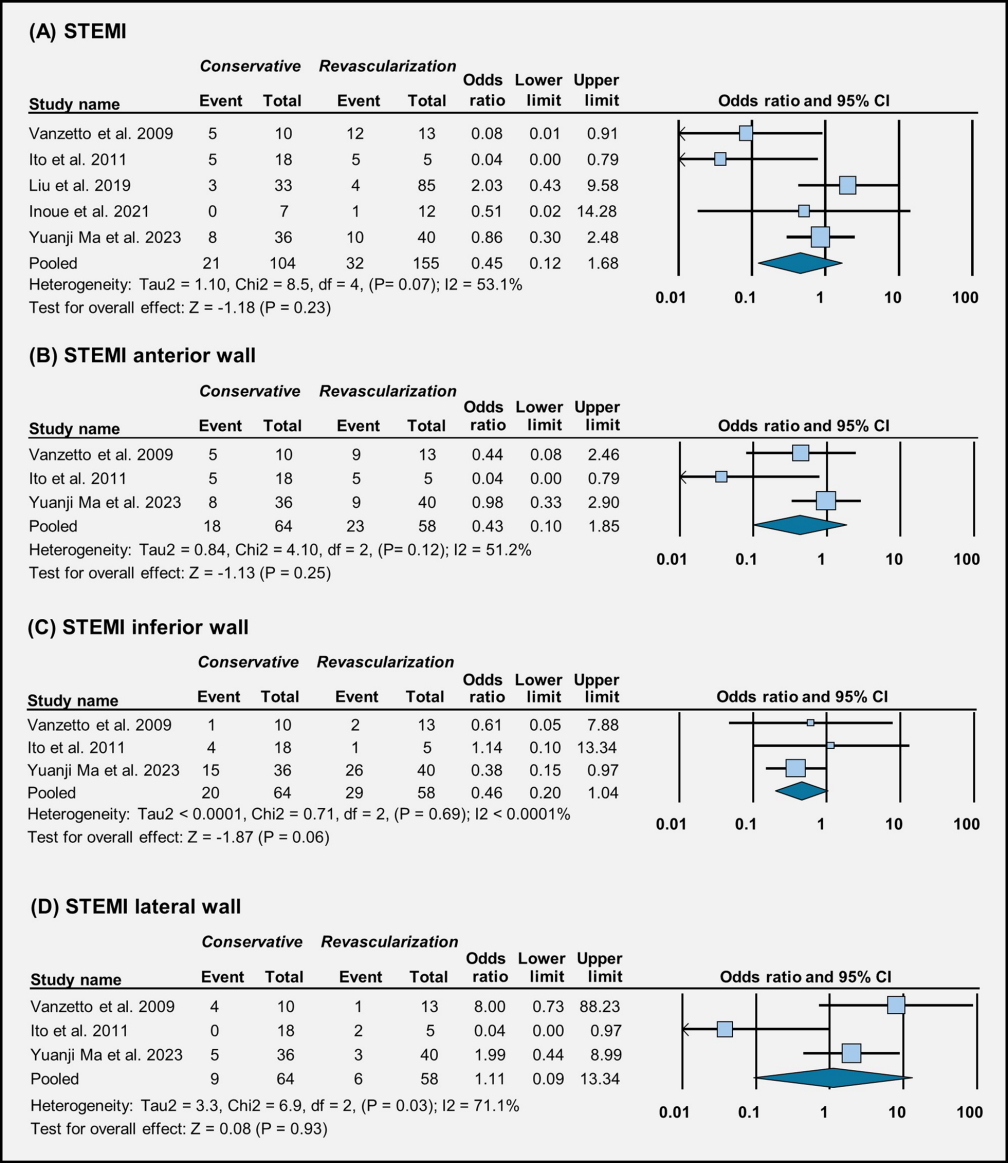
Forest plot illustration of treatment outcomes: **A** STEMI, **B** STEMI anterior wall, **C** STEMI inferior wall, and **D** STEMI lateral wall

Across three studies, the occurrence of stroke does not seem to be affected much by the choice of treatment (OR = 0.35 [95% CI 0.11–1.11], *p* = 0.07) (Fig. 3A). In four studies, we did not observe a significant difference in the occurrence of heart failure between conservative management and revascularization (OR = 0.41 [95% CI 0.09–1.84], *p* = 0.24) (Fig. 3B). Across six studies, in-hospital mortality rates were similar between patients who received conservative management and those who underwent revascularization (OR = 0.35 [95% CI 0.11–1.19], *p* = 0.09) (Fig. 3C). Similarly, across five studies, post-discharge mortality rates did not show much difference between the two treatment groups (OR = 1.66 [95% CI 0.66–4.17], *p* = 0.27) (Fig. 3D). Across five studies, there is no clear difference in the occurrence of STEMI between the two treatment approaches (OR = 0.45 [95% CI 0.12–1.68], *p* = 0.23) (Fig. 4).

### Sensitivity analysis

**Fig. 5:**
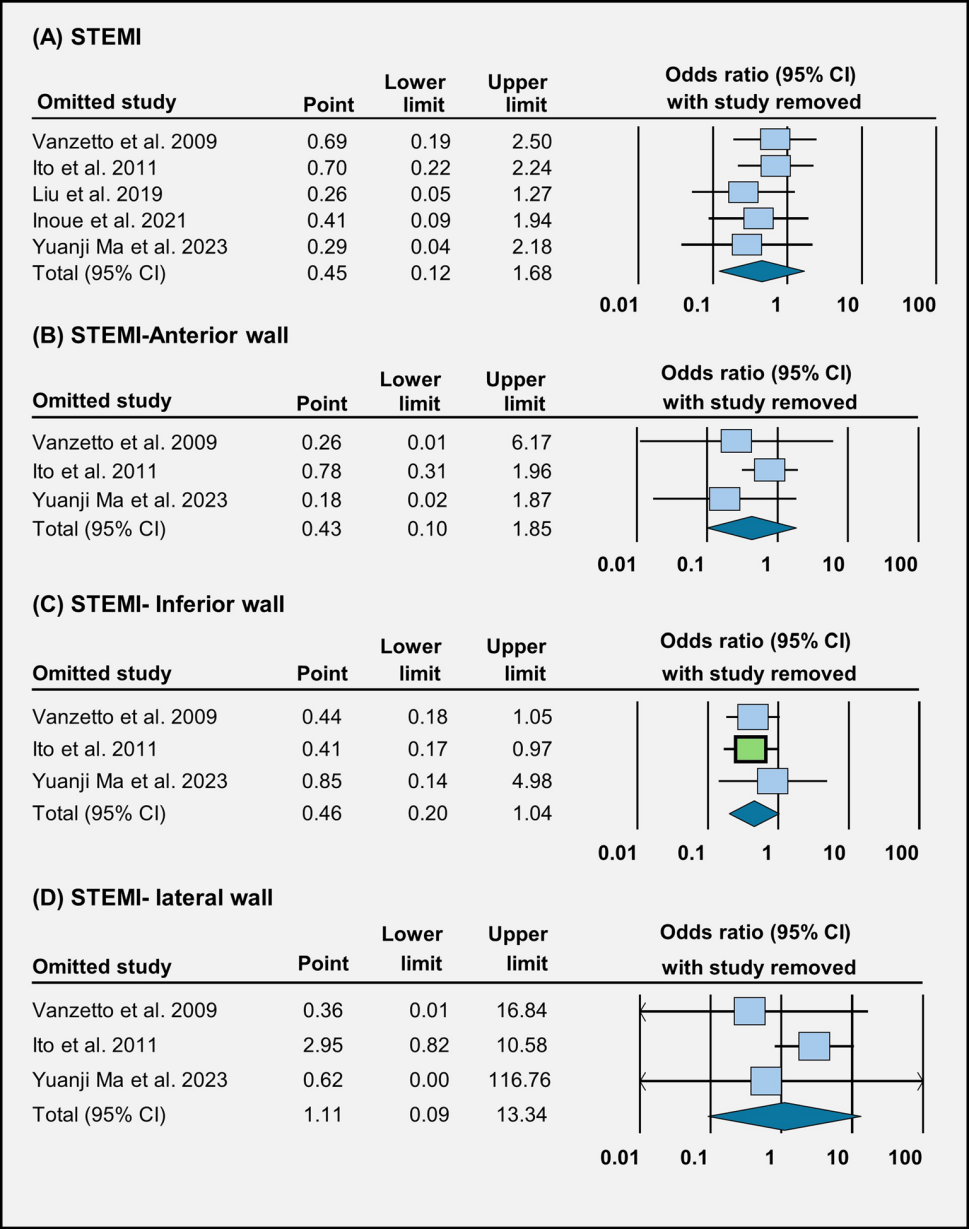
Sensitivity analysis evaluating **A** STEMI, **B** STEMI anterior wall, **C** STEMI inferior wall, and **D** STEMI lateral wall

**Fig. 6:**
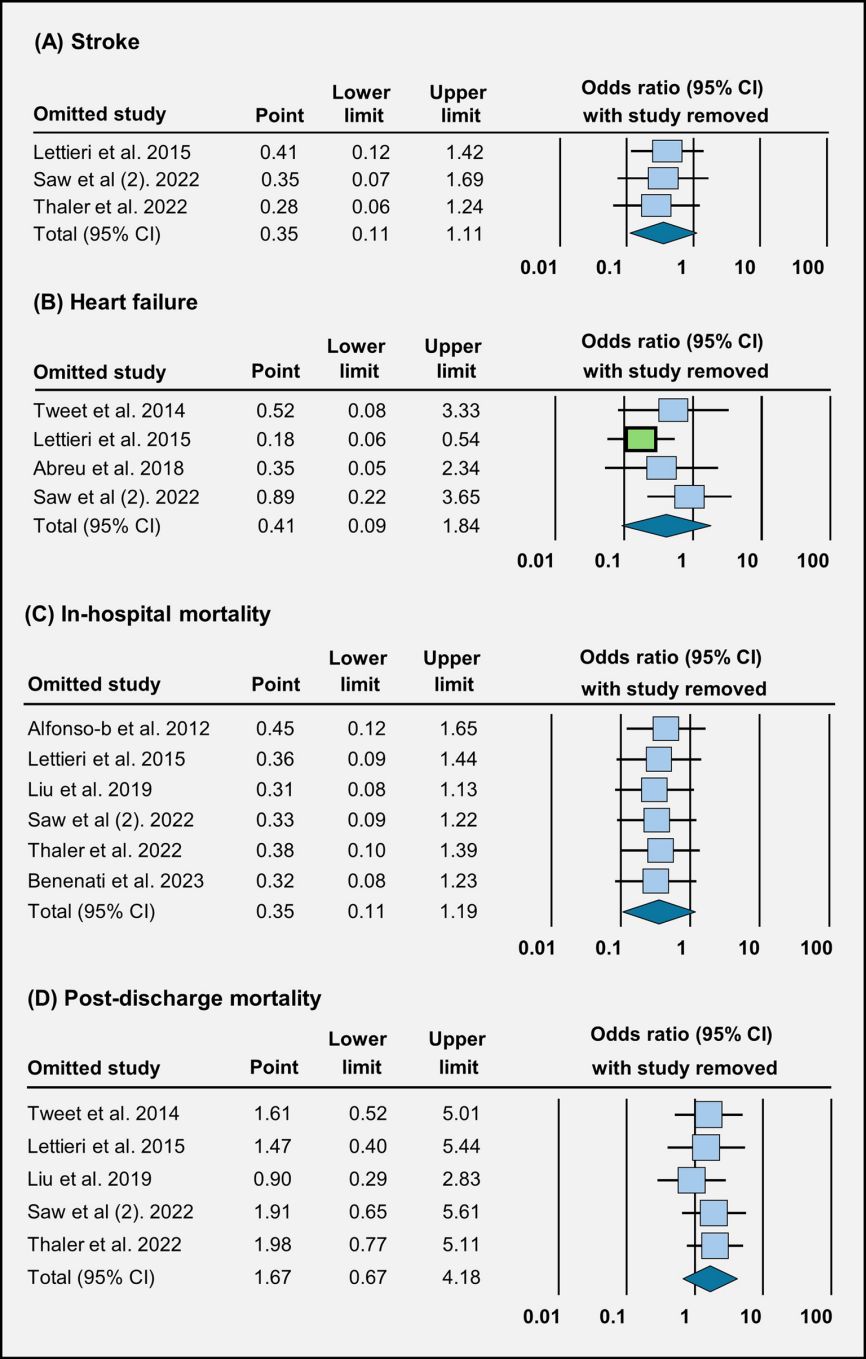
Sensitivity analysis evaluating **A** stroke, **B** heart failure, **C** in-hospital mortality, and **D** post-discharge mortality

**Fig. 7:**
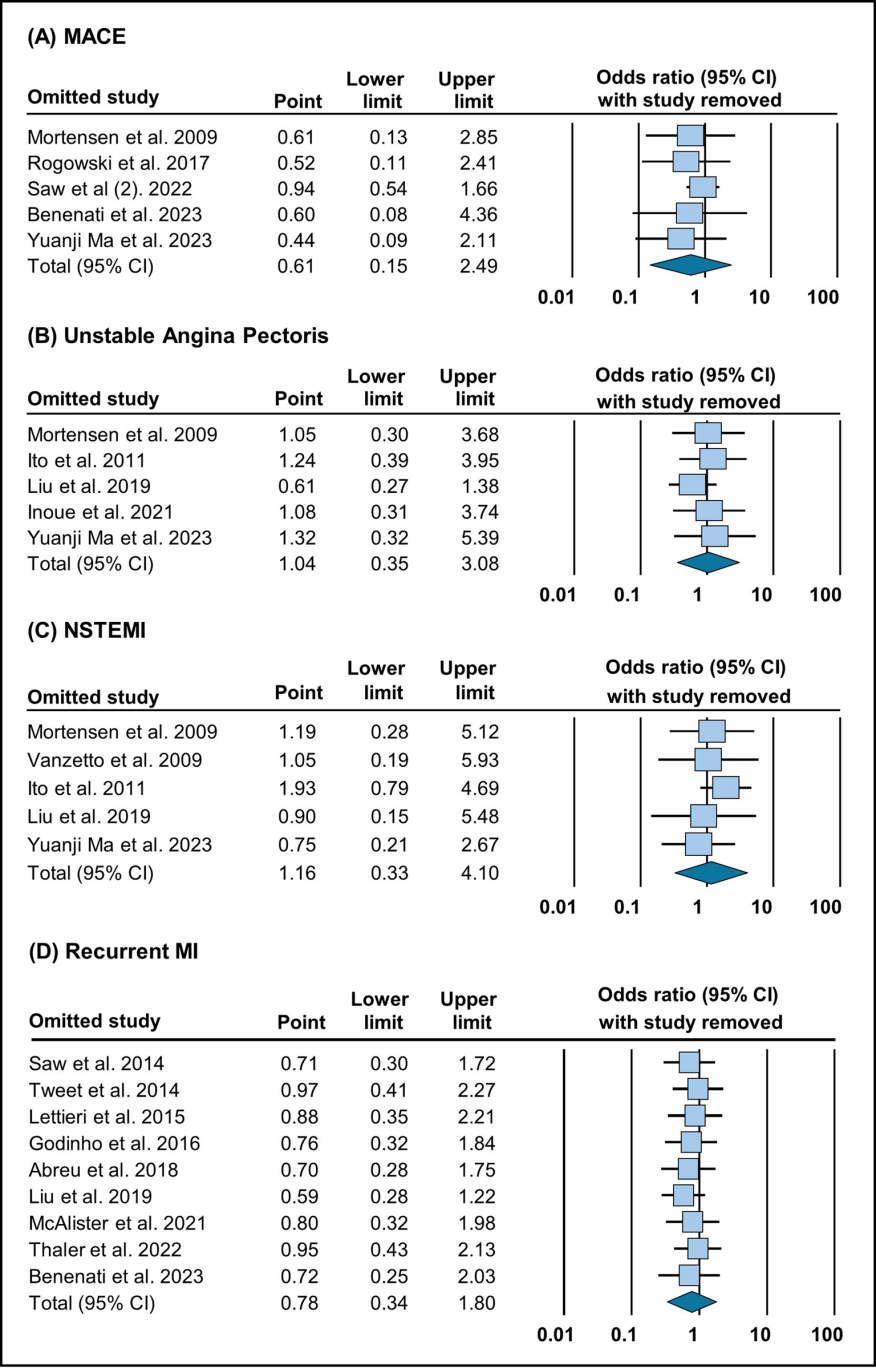
Sensitivity analysis evaluating **A** MACE, **B** unstable angina pectoris, **C** NSTEMI, and **D** recurrent MI

Sensitivity analysis on the removal of Ito et al. showed a significant decrease in odds of inferior wall STEMI in conservative treatment (OR = 0.41 [95% CI 0.17–0.97], *p* = 0.04) compared to revascularization treatment (Fig. 5C). There was also a significant decrease in heart failure in the conservative treatment group (OR = 0.18 [95% CI 0.06–0.54], *p* = 0.002) compared to the revascularization treatment group on the removal of Lettieri et al. [49] (Fig. 6B). Sensitivity analysis on other outcomes does not show significant variation in the outcomes between the two treatment populations (Fig. 7).

### Publication bias

**Fig. 8:**
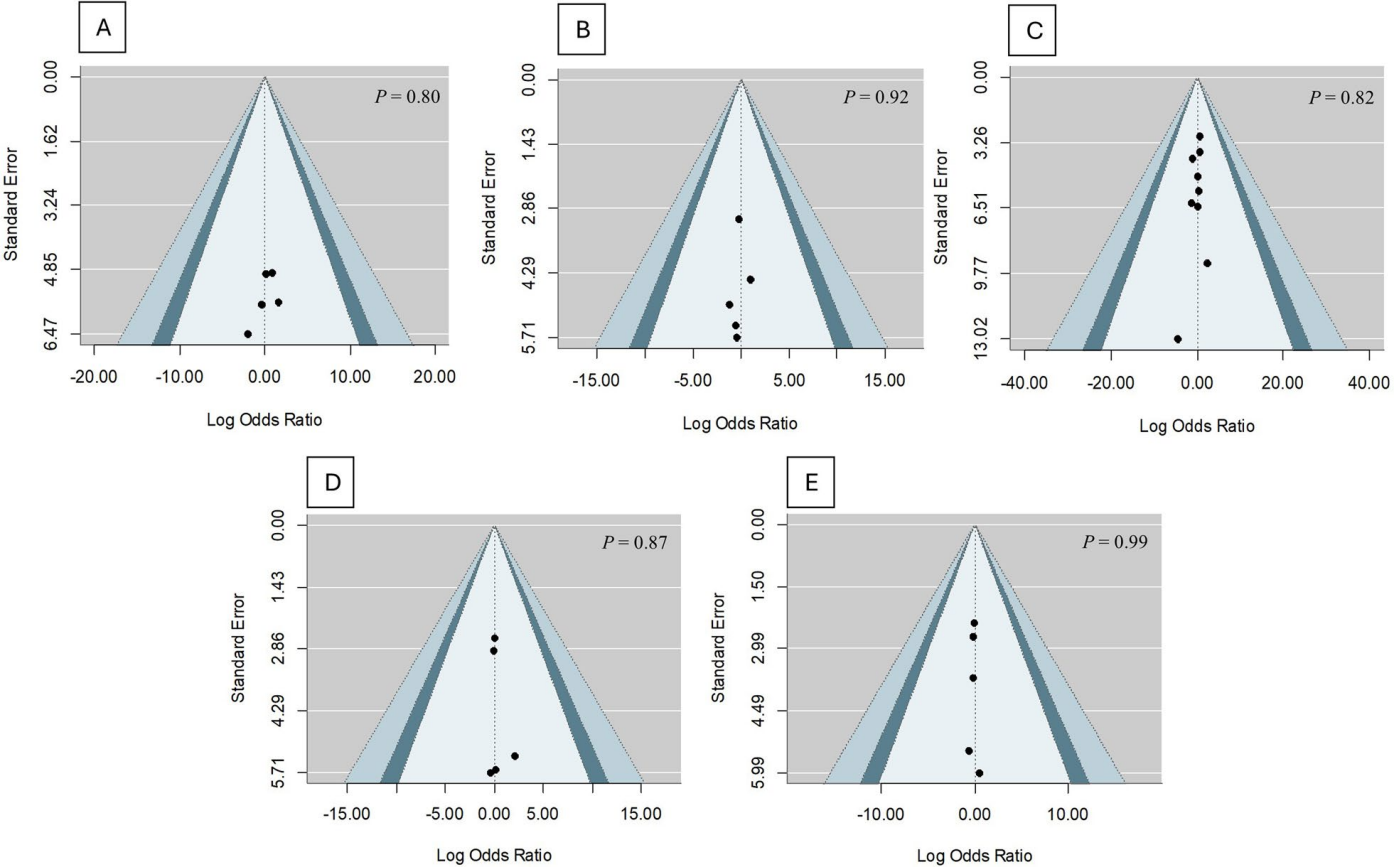
Contour-enhanced funnel plot for **A** MACE, **B** unstable angina pectoris, **C** recurrent MI, **D** NSTEMI, and **E** STEMI

**Fig. 9:**
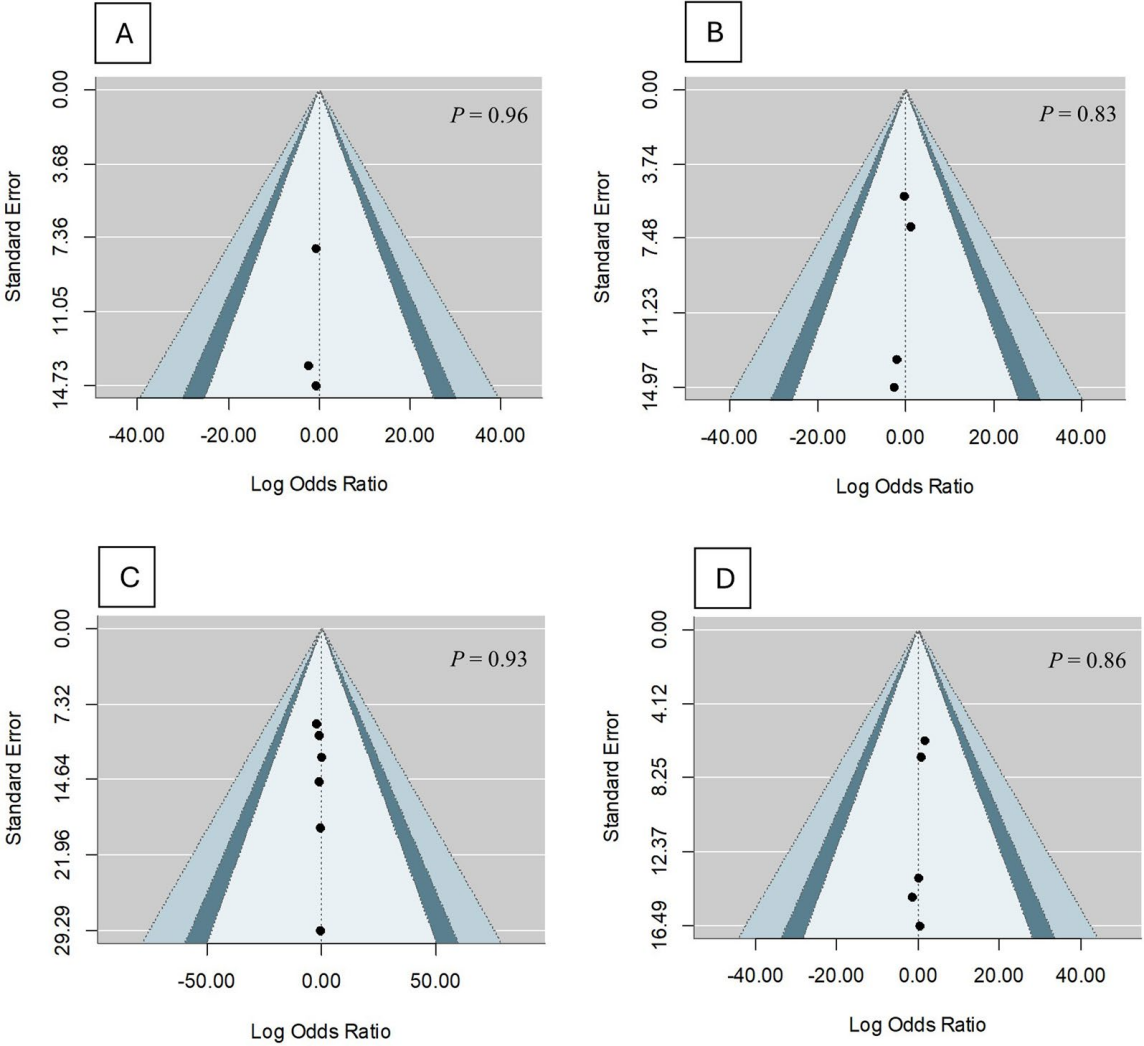
Contour-enhanced funnel plot for **A** stroke, **B** heart failure, **C** in-hospital mortality, and **D** post-discharge mortality

Based on the contour-enhanced funnel plot assessments, no asymmetry was noted in all the outcome variables, signifying the absence of publication bias (Figs. 8 and 9). Eggers' regression, which is given in detail in Table 2, further confirms this. This rules out the possibility of publication bias, further enhancing the stability of our study.

**Table 2:** Egger’s regression analysis to assess publication bias for the outcomes between conservative and revascularization treatments

Outcomes	Effect size (Beta) with 95% CI	Z-value (Egger)	*p*-value (Egger)	Publication bias
MACE	1.7572 (CI: −41.5193, 55.0336)	−0.2691	0.7879	Not significant
Unstable angina	0.5211 (CI: −41.5193, 16.8147)	−0.0882	0.9297	Not significant
Pectoris (UAP)	0.5211 (CI: −15.7725, 16.8147)	−0.0882	0.9297	Not significant
NSTEMI	−0.5151 (CI: −10.1023, 9.0721)	0.1516	0.8795	Not significant
STEMI	−0.1877 (CI: −8.9145, 8.5391)	0.0101	0.9919	Not significant
Recurrent MI	0.9301 (CI: −7.5365, 9.3966)	−0.2185	0.8270	Not significant
Stroke	−0.1296 (CI: −42.8680, 42.6088)	−0.0490	0.9609	Not significant
Heart failure	1.7345 (CI: −17.8968, 21.3659)	−0.2023	0.8396	Not significant
In-hospital mortality	−2.6650 (CI: −34.2590, 28.9289)	0.0832	0.9337	Not significant
Post-discharge mortality	2.5349 (CI: −18.4737, 23.5435)	−0.1675	0.8670	Not significant

## Discussion

There currently exists no randomized controlled trials (RCT) comparing the outcomes of revascularization with a conservative strategy for SCAD patients.

This meta-analysis of clinical outcomes in SCAD indicates that there is no significant difference in MACE between conservative management and revascularization procedures (OR = 0.61 [95% CI 0.15–2.49], *p* = 0.49). Furthermore, the occurrence of UAP remains similar regardless of the treatment approach (OR = 1.04 [95% CI 0.35–3.07], *p* = 0.93). Likewise, the rates of NSTEMI (OR = 1.16 [95% CI 0.33–4.10], *p* = 0.82) and recurrent MI (OR = 0.78 [95% CI 0.33–1.80], *p* = 0.56) do not significantly differ between the two treatment strategies. The occurrence of stroke and heart failure also does not appear to be significantly influenced by the choice of treatment. In-hospital mortality rates were similar between patients who received conservative management and those who underwent revascularization (OR = 0.35 [95% CI 0.11–1.19], *p* = 0.09). Pooled results of this analysis indicate that conservative treatment for SCAD patients resulted in similar in-hospital and long-term clinical outcomes compared to those who underwent revascularization. These findings suggest that both conservative management and revascularization strategies yield comparable clinical outcomes in SCAD patients, highlighting the need for individualized treatment decisions based on patient-specific factors and preferences. Additionally, data from large-scale prospective registries with longer follow-ups are needed to assess whether medicine alone effectively prevents recurrent dissections.

Our findings are comparable to the previous meta-analysis findings by Pitliya et al., Martins et al., Krittanawong et al., and Jamil et al.[71–74]. The analysis by Shamloo et al. revealed that while a conservative management approach initially demonstrated lower rates of target vessel revascularization (TVR), a notable proportion of patients ultimately required surgical or catheter-based interventions [75]. The study's significant shortcomings are selection bias, publication bias, and non-uniform follow-up. The data are mostly based on published case reports, resulting in inherent limitations.75 Bocchino et al., in a meta-analysis, found that a conservative approach for SCAD was associated with a lower TVR rate compared to invasive treatment, while no significant differences were observed in all-cause death, cardiovascular death, myocardial infarction, heart failure, or SCAD recurrence [76]. These results suggest that medical therapy may be appropriate for clinically stable SCAD patients without high-risk features.

SCAD, characterized by non-traumatic dissociation of the coronary vessel wall, poses a significant risk of myocardial infarction and sudden death, particularly among young or middle-aged women lacking typical atherosclerotic risk factors [77]. Historically, pathological studies consistently demonstrated a notable eosinophilic infiltration in the adventitia or periadventitial layer of the coronary artery in SCAD patients.78 This observation suggests a potential causal relationship between eosinophils and SCAD development, supported by their capacity to damage vascular endothelium and induce intramural hemorrhage [77]. Future studies should target considering eosinophilic inflammation to improve the clinical outcome in patients with SCAD. Recent attention has been drawn to the potential association between SCAD and myocardial bridging (MB) [78]. These findings propose mechanistic links such as vasospasm induced by systolic kinking in the bridged artery, endothelial dysfunction associated with MB, and disrupted coronary flow dynamics [78, 79]. This underscores the importance of exploring the mechanistic implications of MB in patients with SCAD.

SCAD typically follows a relatively benign trajectory compared to other causes of ACS, with spontaneous resolution observed in the majority of patients after initial conservative management [47, 51]. However, PCI for SCAD carries a higher risk of complications compared to PCI for atherosclerotic ACS, with as many as 35–53% of SCAD patients experiencing technical failure. Possible causes of procedural failure include inadvertent guidewire passage into the false lumen, propagation of intramural hemorrhage by balloon dilation or stent placement, extension of dissection due to the fragile vessel wall, coronary artery tortuosity, and extensive involvement of distal coronary segments or small side branches [80, 81]. Data from large SCAD cohorts suggest that PCI failures may adversely impact clinical outcomes, with approximately 9–13% of patients requiring emergency CABG due to procedural failures and around 31% undergoing early CABG experiencing late graft closure [31, 40]. Current recommendations from the American Heart Association and the European Society of Cardiology advocate for conservative management in hemodynamically stable SCAD patients without ongoing ischemia, left main coronary dissection, or high-risk anatomical features [31, 80–82].

Innovative techniques have been researched to improve outcomes for SCAD patients. Mele et al. used sirolimus-eluting self-expanding stents (SES) in a male patient with a long dissection lesion in the distal right coronary artery, anticipating to apply less pressure to the vulnerable coronary wall and accommodating a wide range of diameters, potentially reducing trauma and dissection extension while providing sustained expanding force post-intramural hemorrhage reabsorption to minimize malposition and stent thrombosis [83]. Ricci et al., on the other hand, emphasized the importance of imaging techniques, using myocardial perfusion single-photon emission computed tomography and multidetector computerized tomography coronary angiography to monitor perfusion defects and delineate untreated dissections, respectively, to aid decision-making in SCAD patients managed conservatively [84].

The most appropriate medical therapy regimen for SCAD patients remains unknown [31, 52, 85, 86]. In the absence of left main involvement and persistent ischemia, a conservative strategy is typically favored to avoid the risks associated with PCI failure and CABG. Antiplatelet medicines may protect against thrombosis in the prothrombotic environment created by intimal damage and turbulent flow. Beta-blockers may help manage arrhythmia or left ventricular dysfunction and potentially prevent SCAD extension or recurrence by reducing shear stress in the coronary artery [82]. Angiotensin-converting enzyme inhibitors or angiotensin II receptor blockers may be considered for post-ACS patients with systolic dysfunction, whereas statins should only be used for primary prevention of atherosclerosis or in patients with established concomitant atherosclerosis or diabetes mellitus [87, 88]. The role of anti-inflammatory medication in preventing SCAD recurrence is unclear and warrants additional exploration [77]. It is worth noting that the majority of patients (64.4%) got first conservative therapy, suggesting a lower-risk profile than those who received primary PCI or CABG [88]. Prospective investigations comparing outcomes between individuals with similar risk profiles, hemodynamic stability, lesion location, and disease severity are required for the validation of these findings [31, 52, 85, 86].

PCI for SCAD requires careful execution due to potential unsatisfactory outcomes, as found by Tweet et al. and Lettieri et al., which may contribute to greater rates of TVR in the revascularization group [47, 49]. Angiographic assessment may not accurately predict acute success because the amount of the intramural hematoma may be overestimated, resulting in unexpected flow loss after stent implantation [47, 89]. PCI involves several challenges, such as trouble accessing the actual lumen, the possibility of iatrogenic dissection, and the possibility of a spreading hematoma during angioplasty or stenting that could impair arterial flow [40]. Novel approaches, such as cutting balloon angioplasty and intravascular imaging-guided PCI, may reduce these risks [2, 62, 89]. Revascularization did not reduce long-term TVR risk, demonstrating the importance of diligent follow-up [89]. These findings support a conservative approach, as there is no prognostic difference between medical and revascularization strategies [47–49, 89].

## Limitations

The limitations and differences of the original studies that were part of the analysis will affect the results of this meta-analysis. The meta-analysis has limitations since it only includes retrospective studies with small sample sizes and long-term survival outcomes. The first therapeutic strategy for SCAD was decided by the treating physician, and none of the included studies was a randomized trial. Patients who had medical therapy may have demonstrated greater stability and fewer complications than those who underwent revascularization. The approach may vary across centers and over time due to advancements in technology and equipment, creating a selection bias. The data on anatomical aspects (location and amount of dissection), pharmacologic agents (regimen, dosage, and duration of drugs), and clinical factors (hemodynamic stability, and continued ischemia) were not generally accessible. Additionally, the duration of follow-up was variable among the included studies and might have been insufficient to capture long-term outcomes accurately. Despite efforts to identify all relevant studies, the possibility of publication bias cannot be completely ruled out. The findings of the meta-analysis might not apply to all SCAD patients, as the included studies predominantly focused on specific populations or settings. Factors such as comorbidities, concomitant medications, and procedural techniques might have varied across the included studies, potentially confounding the results.

## Conclusion

Pooled data suggest in the absence of persistent ischemia or left main artery involvement, conservative, and revascularization techniques can yield comparable in-hospital and long-term outcomes under the present decision-making frameworks. Conservative treatment of inferior wall STEMI and heart failure dramatically reduced these outcomes as compared to revascularization in SCAD treatment. Overall, while there were no significant changes in cardiovascular outcomes between the two strategies, these sensitivity findings illustrate the potential advantages of conservative care. Additional data from prospective studies are needed to validate these conclusions.

## Supplementary Information

Supplementary file 1

## Data Availability

The datasets used and/or analyzed during the current study are available from the corresponding author on reasonable request.

## Abbreviations

SCAD: Spontaneous coronary artery dissection
NOS: Newcastle–Ottawa Scale
JBI: Joanna Briggs Inventory
CMA: Comprehensive meta-analysis
MACE: Major adverse cardiac events
UAP: Unstable angina
MI: Myocardial infarction
NSTEMI: Non-ST segment elevation myocardial infarction
STEMI: ST segment elevation myocardial infarction
MI: Myocardial infarction
IMH: Intramural hematoma
ACS: Acute coronary syndrome
PCI: Percutaneous coronary intervention
CABG: Coronary artery bypass grafting
PRISMA: Preferred Reporting Items for Systematic Reviews and Meta-analysis
OR: Odds ratio
CI: Confidence interval
RCTs: Randomized controlled trials
TVR: Target vessel revascularization
MB: Myocardial bridging
SES: Self-expanding stents
