# Supplementary material for: In-hospital and long-term clinical outcomes of spontaneous coronary artery dissection (SCAD): a meta-analysis of conservative versus revascularization approaches
Source: Egypt Heart J. 2024 Nov 22;76:153. doi: 10.1186/s43044-024-00585-0 (PMC11584847; doi:10.1186/s43044-024-00585-0)
Supplement: Supplementary file 1 [file 43044_2024_585_MOESM1_ESM.docx]

**Table: Quality assessment of Case-control studies in New-Castle Ottawa Scale**

| **First author** | **Year of publication** | **Selection** | **Comparability** | **Exposure** | **Total** |
| --- | --- | --- | --- | --- | --- |
| Chen [29] | 2021 | ** | * | ** | 5 |
| Adams [53] | 2017 | ** | ** | ** | 6 |

**Table: Quality assessment of Cohort studies in New-Castle Ottawa Scale**

| **First author** | **Year of Publication** | **Selection** | **Comparability** | **Outcome** | **Total** |
| --- | --- | --- | --- | --- | --- |
| Mahmoud [55] | 2018 | **** | ** | *** | 9 |
| Mortensen [17] | 2009 | *** | - | *** | 6 |
| Ito [46] | 2011 | **** | * | *** | 8 |
| Alfonso-b [30] | 2012 | *** | - | * | 6 |
| Seidl [64] | 2021 | **** | - | *** | 7 |
| Saw (4) [31] | 2014 | **** | - | *** | 7 |
| Tweet (1) [47] | 2014 | **** | * | *** | 8 |
| Lettieri [49] | 2015 | **** | * | *** | 8 |
| McGrath-Cadell [28] | 2016 | *** | - | *** | 6 |
| Nakashima [9] | 2016 | *** | - | *** | 6 |
| Lobo [50] | 2017 | **** | * | **** | 9 |
| Rogowski [46] | 2017 | *** | - | *** | 6 |
| Saw (1) [52] | 2017 | *** | - | *** | 6 |
| Saw (2) [62] | 2022 | *** | * | *** | 7 |
| Saw (3) [56] | 2019 | *** | - | **** | 7 |
| Clare [10] | 2019 | **** | ** | *** | 9 |
| Daoulah [11] | 2021 | **** | ** | *** | 9 |
| Salamanca [63] | 2023 | **** | - | *** | 7 |
| Díez-Villanueva [57] | 2021 | **** | - | *** | 7 |
| Tweet (2) [40] | 2012 | **** | ** | *** | 9 |
| Liu [20] | 2019 | *** | - | *** | 6 |
| Benenati [68] | 2023 | **** | - | *** | 7 |
| McAlister [58] | 2021 | *** | - | ** | 5 |
| Wilander [65] | 2022 | *** | - | *** | 6 |
| Thaler [66] | 2022 | **** | - | *** | 7 |
| Proença T [69] | 2023 | *** | - | *** | 6 |
| [García-Guimaraes](https://pubmed.ncbi.nlm.nih.gov/?term=Garc%C3%ADa-Guimaraes+M&cauthor_id=32418854) [59] | 2021 | *** | - | *** | 6 |
| Ma [66] | 2023 | **** | * | *** | 8 |
| Sharma [54] | 2017 | **** | * | *** | 8 |
| Inoue [24] | 2021 | *** | * | *** | 7 |

**Table: Quality assessment of Cohort studies in Joanna Briggs Inventory Scale**

| **First author** | **Year of publication** | **Total** | **Interpretation** |
| --- | --- | --- | --- |
| Vanzetto [18] | 2009 | 100% | Good |
| Kansara [36] | 2011 | 90% | Good |
| Alfonso-a [37] | 2012 | 100% | Good |
| Sultan [39] | 2015 | 100% | Good |
| Roura [40] | 2016 | 90% | Good |
| Cade [43] | 2017 | 100% | Good |
| Abreu [44] | 2018 | 100% | Good |
| Inohara [45] | 2020 | 100% | Good |
| Rashid [42] | 2016 | 100% | Good |
| Chan [67] | 2022 | 100% | Good |
| Motreff [35] | 2010 | 100% | Good |
| Manhaes [38] | 2014 | 90% | Good |
| Godinho [41] | 2016 | 90% | Good |
| De Roeck [60] | 2021 | 100% | Good |
